# Ultra-Long-Term Therapy of Benign Essential Blepharospasm with Botulinumtoxin A—30 Years of Experience in a Tertiary Care Center

**DOI:** 10.3390/toxins14020120

**Published:** 2022-02-07

**Authors:** Bettina Wabbels, Rolf Fimmers, Peter Roggenkämper

**Affiliations:** 1 Department of Ophthalmology, University Hospital of Bonn, Ernst-Abbe-Street 2, D-53127 Bonn, Germany; proggenk@uni-bonn.de; 2 Institute of Medical Biometry, Informatics and Epidemiology, University Hospital of Bonn, D-53127 Bonn, Germany; fimmers@ifsq.de

**Keywords:** blepharospasm, botulinum toxin, long-term, outcome, adverse events

## Abstract

Aim of this study was to investigate the long-term results of botulinum toxin A (BoNT-A) injections for the treatment of benign essential blepharospasm (BEB) and to report our experience with (ultra-)long-term treatment with onabotulinumtoxin-A. We conducted a retrospective cross-sectional analysis at a university hospital. Patients with BEB and BoNT-A treatment were assigned to the Total Blepharospasm Group, patients with ≥21 onabotulinumtoxin-A injections to the Ona Long-Term Group. The Total Blepharospasm Group (*n* = 1940) included 33,933 BoNT-A injections. The age of patients at symptom onset was (mean ± SD) 58.0 ± 13.1 years, and 70.4% were female. The Ona long-term group (*n* = 234) included 10,632 onabotulinumtoxin-A injections. In this group, patients received 45.4 ± 22.9 injections with a mean dose of 22.2 IU ± 0.5. The duration of treatment was 12.6 ± 5.4 years, ranging from 2.9 to 30.0 years. The effect–duration–dose quotient did not change during long-term treatment. The observed side effects were comparable in type and frequency to other studies, even with the (ultra-)long treatment with onabotulinumtoxin-A. Our results, based on one of the largest patient populations and a treatment duration of up to 30 years, impressively demonstrate that onabotulinumtoxin-A is a safe and effective therapy for essential blepharospasm, even in the ultra-long term.

## 1. Introduction

Benign essential blepharospasm (BEB) is a focal cranial dystonia characterized by chronic, intermittent, or persistent involuntary eyelid closure due to spasmodic contractions of the orbicularis oculi muscles. The condition usually affects both eyes, with severity ranging from repetitive frequent blinking to persistent severe eyelid closure leading to functional blindness [1]. Blepharospasm occurring in combination with oromandibular dystonia (involuntary spasms of the tongue, jaw, throat, and face) is referred to as Meige syndrome [2]. Usually, the initial manifestation of BEB occurs in the fifth decade of life, with women more commonly affected than men by a ratio of 2:1 [3]. The prevalence of BEB has a high regional variance (ranging from 16 to 133 cases per 1,000,000 persons) and is approximately 36 per 1,000,000 in Europe [4,5].

In the 1980s, botulinum toxin type A (BoNT-A), a potent exotoxin produced by Clostridium botulinum, was shown to be an effective treatment for BEB [6,7,8,9]. Its effect occurs via inhibiting acetylcholine release at the neuromuscular junction, resulting in decreased muscle contraction [10]. Symptom reduction can be achieved in approximately 90% of BoNT-A-treated patients with BEB. A systematic review according to evidence-based medicine criteria concluded that BoNT-A can be considered a first-line therapy for primary cranial dystonia with a recommendation grade A [11]. In case of non-response to botulinum injections, suspension surgery is another therapeutic option to improve functionality and reduce the frequency of spasms [12].

In Europe and the United States, three BoNT-A products—onabotulinumtoxin A (BOTOX^®^, Allergan plc, Dublin, Ireland), incobotulinumtoxin A (XEOMIN^®^, Merz GmbH & Co., Frankfurt, Germany), and abobotulinumtoxin A (Dysport^®^, Ipsen, Slough, Berkshire, UK)—are commercially available for therapeutic use in BEB. In order to maintain improvement, repeated BoNT-A injections have to be performed on a regular basis. In general, BoNT-A injections are recommended every three to four months [12]. Given the required long-term use, many patients raise concerns about a diminishing treatment effect and increasing adverse effects. Some authors report a stronger effect with the first round of injections and diminishing efficacy with subsequent injections [13].

Although there are many reports of short-term efficacy of various types of BoNT-A for the treatment of BEB, long-term studies with large patient collectives on the efficacy and safety of BoNT-A in BEB are limited [14,15,16,17,18,19]. At the University Eye Hospital in Bonn, Germany, BoNT-A was used for the treatment of BEB since 1985 and therefore, long-term data are available for a large patient population.

The aim of the present study is to retrospectively investigate the (ultra-)long-term outcomes of BoNT-A treatment of patients with BEB in terms of dosage, stability of effect duration, and adverse effects during long-term treatment and to report our experience with long-term treatment with BoNT-A.

## 2. Results

### 2.1. Patient Demographics and Baseline Data

A total of 3304 patient records on BoNT-A injections for various indications (BEB, hemifacial spasm, and less frequently synkinesia after facialis paresis, strabismus, crocodile tears, protective ptosis and Graves’ disease) were screened. This retrospective analysis included the medical records of 1940 patients who had received 33,933 BoNT-A injections for BEB treatment since 1985 (Total Blepharospasm Group). Depending on individual patient aspects and availability of the respective drug, patients were treated with onabotulinumtoxin A, incobotulinumtoxin A, and abobotulinumtoxin A. In the Total Blepharospasm Group, the mean age of patients at the onset of symptoms was 58.0 ± 13.1 years, and 70.4% were female. Patients first presented to our clinic at a mean age of 63.3 ± 11.9 years and had received BoNT-A treatment for an average of 4.9 ± 6.2 years, with a maximum duration of 30 years.

Patients with at least 21 BoNT-A injections were defined as long-term patients. As most of these patients had received onabotulinumtoxin A in at least 95% of injections, detailed analyses of BoNT-A long-term treatment were performed with respect to this homogeneous onabotulinumtoxin A long-term group (Ona Long-Term Group; *n* = 234). Table 1 summarizes the demographics and treatment-associated parameters of the Total Blepharospasm Group and the Ona Long-Term Group. In terms of demographics, the two groups compare well, with a slightly higher proportion of women and patients with frontalis-suspension in the Ona Long-Term Group.

### 2.2. Onabotulinum A Treatment in the Long-Term Course

**Figure 1:**
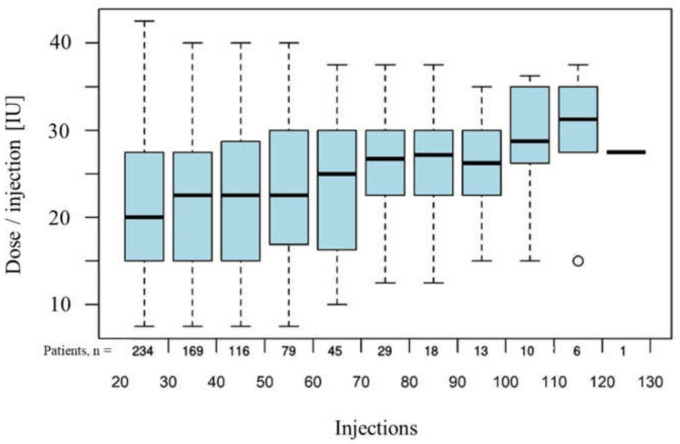
Doses of onabotulinumtoxin A administered over the long term, with time represented by consecutive number of injections. The box of a box-plot represents the quartiles (25%- and 75% percentile) of the empirical distribution. The wiskers show values up to the 1.5 fold of the inquartile range above and below the box. Values outside this range are represented by circles.

On average, patients in the Ona Long-Term Group received 45.4 ± 22.9 onabotulinumtoxin A injections (range 21–153) over a treatment period of 12.6 ± 5.4 years (range: 2.9 to 30.0 years), corresponding to 10,632 injections of onabotulinumtoxin A. A mean dose of 22.2 IU ± 0.5 (SEM, 95% CI: (21.3, 23.1)) was administered per injection to each side of the face. Over the long-term, an increase in the dose of 0.042 (95% CI: (0.031, 0.54)) IU per injection was observed (Figure 1). Dose data were not available for one patient.

**Figure 2:**
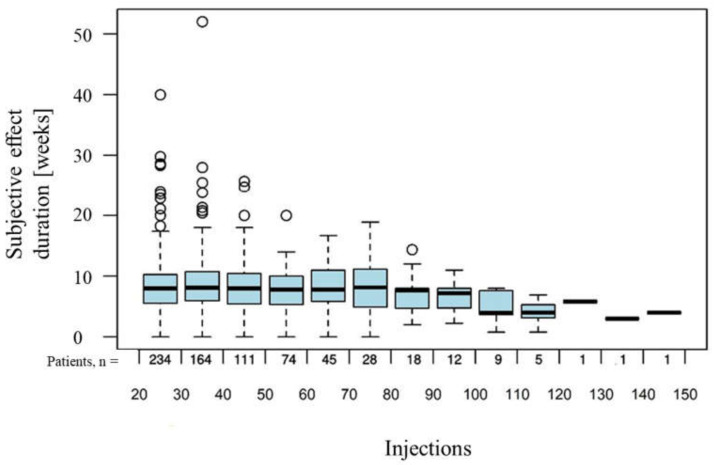
Subjective effect duration of onabotulinumtoxin A administered over the long term, with time represented by consecutive number of injections. Values outside the 1.5 fold of the inquartile range above the box range are represented by circles.

The mean subjective effect duration of all onabotulinumtoxin A injections was reported to be 8.8 ± 0.3 (SEM) weeks per injection. Over the long-term course of onabotulinumtoxin A treatment, the subjective effect duration remained stable, with a slight intraindividual increase of 0.0113 (95% CI: (0.0061, 0.0164)) weeks per injection (Figure 2).

**Figure 3:**
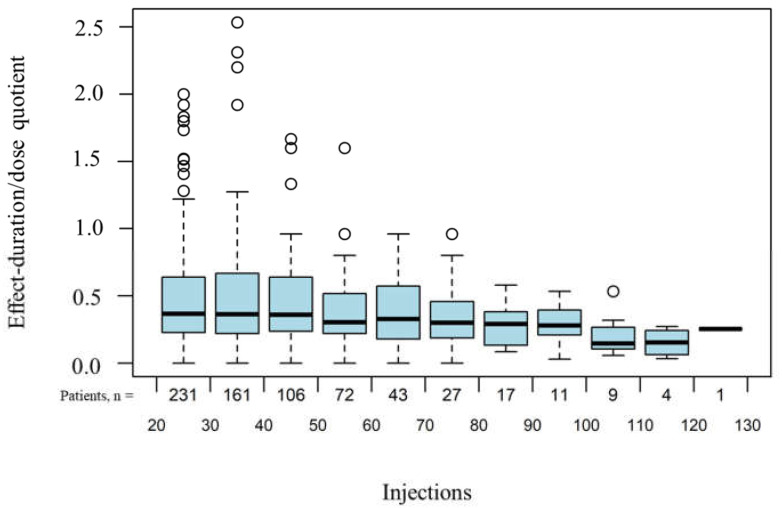
Effect–duration quotient under long-term treatment with onabotulinumtoxin A. Time is represented by consecutive number of injections. Values outside the 1.5 fold of the inquartile range above the box range are represented by circles.

The effect duration-dose quotient per injection did not change during long-term treatment with onabotulinumtoxin A (Figure 3).

In relation to all onabotulinumtoxin A injections administered within the Ona Long-Term Group, the following adverse events were reported: ptosis (2.9%), double vision (2.2%), lacrimation (7.2%), hematoma (3.1%), discomfort (3.1%), and general unspecific symptoms (2.4%).

**Figure 4:**
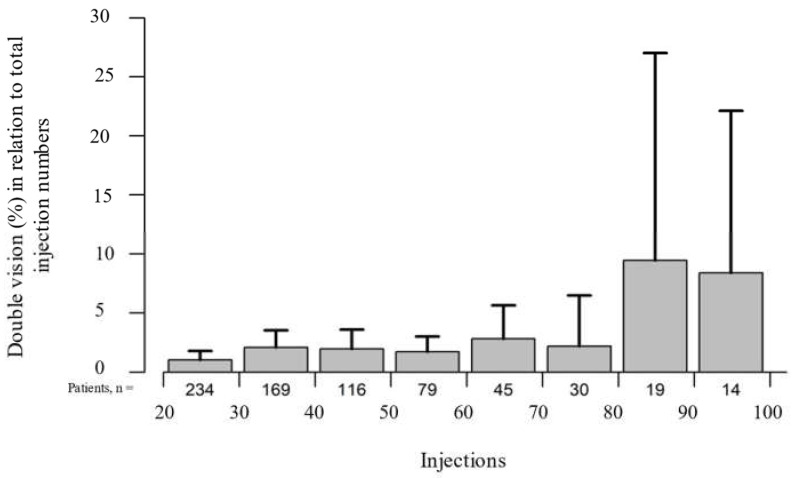
The percentage of double vision in relation to all 10,632 onabotulinumtoxin A injections administered over the long-term course increased significantly (*p* < 0.001). Time is represented by consecutive injection numbers.

**Figure 5:**
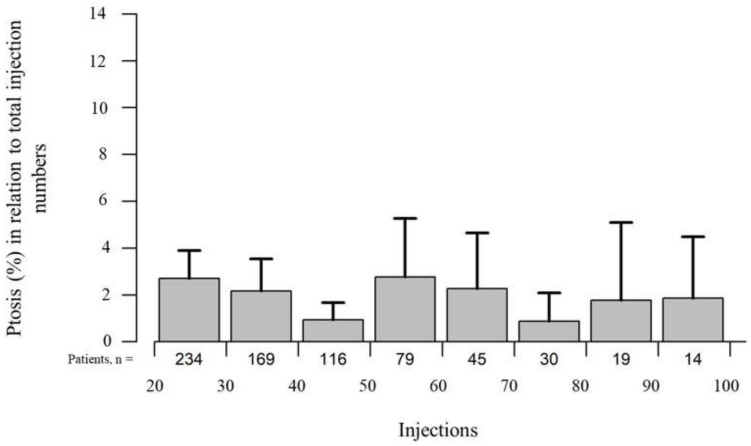
The percentage of ptosis in relation to all 10,632 onabotulinumtoxin A injections administered over the long-term course remained stable (*p* = 0.567). Time is represented by consecutive injection numbers.

In the long-term course, the occurrence of hematoma (*p* = 0.963), discomfort (*p* = 0.350), and general nonspecific symptoms (*p* = 0.714) remained stable. Of the toxin-related side effects, lacrimation and double vision increased significantly (*p* < 0.001) (Figure 4) during long-term treatment with onabotulinumtoxin A, while ptosis remained stable (*p* = 0.567) (Figure 5). No case of complete or visually inhibiting ptosis occurred. None of the observed adverse effects were life-threatening or resulted in treatment discontinuation.

## 3. Discussion

The present study represents one of the most comprehensive surveys of BoNT-A therapy for the treatment of BEB. Although some long-term studies are found in the literature, these analyses usually cover shorter observation periods and smaller patient collectives. A comparison with published studies demonstrates that the present study, with a total of 1940 patients treated for BEB with botulinum toxin A, of whom 234 received long-term treatment with onabotulinum toxin A, represents the largest patient population investigated, with the highest number of injections administered, and with the longest treatment period of up to 30 years (Table 2) [14,16,19,20]. Another publication on long-term use of BoNT-A by Badarny et al. was not included in the table below because of a lack of comparability, as this analysis was performed only for a group of patients, which also included 42 patients with hemifacial spasm and 19 with facial synkinesis in addition to the 26 patients with BEB. Furthermore, patients were treated with either Botox or Dysport, depending on availability. Only eight patients were treated with Botox alone, and no data on subjective duration were available [20].

Another important aspect of our study is the standardized documentation and uniform treatment of all patients following the same injection regimen over the entire course of treatment. Although the injection points were also individually adjusted to the patient—which is essential for successful treatment—they were administered to the respective patient following a consistent regimen from first injection onwards and documented in a standardized manner over many years. Moreover, subjective effect duration and adverse effects were explicitly queried and documented at each visit.

### 3.1. Patient Population

The large patient population included in this study corresponds to a typical population of BEB patients. The higher proportion of females of over 70% and the age of 58 years at the first manifestation of BEB reflect the findings of other studies and are consistent with previous epidemiological findings in the literature [16,19]. The proportion of BEB patients with additional oromandibular dystonies (Meige syndrome) of 30.5% in the present study falls within the range reported in the literature of 23% in Huang et al. and 39.2% in Aquino et al., with the higher proportion reported in Aquino et al. possibly being attributable to the additional inclusion of craniocervical dystonia [21,22].

In addition, the present study shows good agreement between the Total Blepharospasm Group and the Ona Long-Term Group in terms of demographic data and baseline parameters of the disease. Although the proportion of women in the long-term group is higher, this can probably be explained by the higher life expectancy of women. The proportion of patients with frontalis suspension surgery is also higher in the long-term group. This might be due to the fact that patients with severe BEB may show better treatment adherence, are more likely to continue their long-term treatment in an ophthalmic clinic, and, at the same time, require frontalis suspension more frequently during the course of therapy. Overall, the patient population analyzed in the present study is comparable to that of other studies and can be considered suitable for collecting relevant data on BoNT-A therapy for BEB.

### 3.2. Long-Term Treatment with Onabotulinumtoxin A

Our data on the (ultra-)long-term course with up to 153 consecutive injections show that onabotulinumtoxin A treatment can be performed safely and without loss of effect over a long period of up to 30 years: with regard to the administered dose, only a slight increase was observed, and both the subjective effect duration and the effect duration–dose ratio remained stable in the course of long-term treatment.

On average, a mean total dose of 44.4 IU onabotulinumtoxin A (both sides of the face) was applied in the Ona Long-Term Group in the present study. Comparable dosages are described in the literature, e.g., 41 IU in patients of the onabotulinumtoxin A group in the study by Kollewe et al. [16]. Although a lower dose of 34 IU was administered in the onabotulinumtoxin A group in the study by Bentivoglio et al., it has to be taken into account when interpreting the findings that the numbers of onabotulinumtoxin A injections in these studies differed considerably (current study *n* = 10,632, Kollewe et al. *n* = 4974; Bentivoglio et al. *n* = 1009) [16,19]. Ababneh et al. reported a total dose of 46 IU at the beginning of BEB therapy and 52 IU for the last injections at the end of the study, but did not report total injection numbers for BEB patients [14].

Furthermore, our results show, in agreement with most literature data, that the administered dose remains almost stable over the long-term course, with only a slight increase of 0.042 IU per injection. It should be noted, however, that in the present long-term study—unlike in many other studies—the time course of the dosage was analyzed after the 20th injection in order to exclude any influence of the titration phase on the results on the dose in the long-term course. However, definitions of “long-term” vary in the literature. Whereas Bentiviglio et al. included patients with 2 and more BoNT-A injections, Ababneh et al. included patients with a treatment duration of more than 10 years and at least one annual injection, while Kollewe et al. defined more than 8 injections as long-term treatment [14,16,19].

With regard to the mean subjective effect duration, the current evaluation of the Ona Long-Term Group shows slightly lower values compared with other analyses. Compared to the mean subjective effect duration of 8.8 weeks in the Ona Long-Term Group, Kollewe et al. report a mean subjective effect duration of 10.8 weeks [16], while Bentiviglio et al. report 9.5 weeks [19]. However, varying definitions of subjective effect duration as well as individually different assessments by the patients may have contributed to this. While in the present study, the subjective duration of effect refers to the time until the first recurrence of symptoms, in some other studies, it refers to the duration until the absence of any effect. Moreover, it should be noted that our clinic is a major reference center for the treatment of BEB and that many patients, especially those who are difficult to treat externally, are referred to our clinic for therapy adjustment. Subsequently, those patients whose therapy is successfully adjusted are often followed up externally again, while more severe cases often remain at our clinic. It is, therefore, possible that the proportion of patients who are difficult to treat is somewhat higher at our clinic, especially among the long-term patients, which might explain the somewhat shorter subjective effect duration. In the Total Blepharospasm Group, the subjective duration of effect was higher, with 10.3 ± 12.1 weeks. Overall, in agreement with most other studies, our study showed a stable subjective duration of effect with no significant decrease over time, suggesting that patients and physicians do not have to be concerned about an increase in the injection frequency required, even with long-term therapy.

Furthermore, the effect duration–dose quotient was investigated in the present study, as described in the studies by Bedar as well as Nüßgens and Roggenkämper [23,24]. In all three studies, the effect duration–dose quotient also remained stable over time. Overall, the findings allow the conclusion that onabotulinumtoxin A remains effective in the long term and can be administered over a longer period of time.

### 3.3. Adverse Events during Long-Term Treatment

With regard to adverse events, the present study supports literature data showing that side effects are rare even in the long-term course and that treatment with onabotulinumtoxin A can be considered safe. The spectrum of side effects observed in the Ona Long Term Group was comparable to that observed in other studies in terms of type and frequency. No “new” side effects were observed in the (ultra-) long term course of onabotulinumtoxin A therapy.

Consistent with other studies, ptosis was also the most common toxin-related adverse event observed in our analysis and remained stable over the long term. While ptosis was reported more frequently in the studies by Cilino et al. (19.2%) [25], Jankovic et al. (19.19%) [26], and Bentivoglio et al. (9.7%) [19] compared to our study, Kollewe et al. provided comparable results (2.4%) [16].

Double vision is described in the literature as a transient side effect after botulinum toxin injections [9,14,20,25], and we are not aware of any studies describing an increase in this adverse event in the (ultra-) long term. In addition, in our study, double vision remained stable at a low level over the course of up to 80 injections, with a marked increase only thereafter. However, due to the considerably lower number of patients with high injection numbers (Figure 4), this is expected to be a randomly observed effect.

During long-term therapy, a statistically significant increase in lacrimation was observed. However, it should be remembered that with increasing age, incomplete eyelid closure and dry eye also occur more frequently. Both may be accompanied by increased lacrimation, making it difficult to distinguish to what extent the increase in lacrimation was toxin-induced or age-related. Other side effects relevant to the patients, such as hematoma, discomfort, and general unspecific symptoms, remained stable over the course of long-term treatment.

### 3.4. Limitations

As is common in most long-term studies, the patients in this (ultra-)long-term study were treated by different physicians. This may lead to interindividual differences in treatment, although these are likely to be very small due to the standardized approach at our clinic. Furthermore, the present study did not explicitly investigate whether treatment failure is due to antibody production. However, literature data indicate that antibody production during BEB therapy is rather rare. Finally, adding an evaluation scale for treatment efficacy, such as the Global Clinical Improvement-Index (GCI) or specified blepharospasm scales [27], to the standardized documentation form could help to further refine the assessment of treatment efficacy in the future. These scales are actually used for blepharospasm patients in our department for a couple of years, including clinical and quality of life parameters.

## 4. Conclusions

Overall, our results, based on one of the largest patient collectives and a maximum treatment duration of 30 years, impressively demonstrate that onabotulinumtoxin A is a safe and effective therapy for essential blepharospasm, even in the ultra-long term.

## 5. Materials and Methods

Overall, 3304 medical records of patients who had received BoNT-A injections for therapy of different indications at the University Eye Hospital, Bonn, Germany, since 1985 were screened, and data from patients with BoNT-A injections for the treatment of BEB were included in this retrospective cross-sectional analysis (Total Blepharospasm Group). Since BoNT A injections are the gold standard of therapy for BEB, all patients with relevant essential blepharospasm are treated with BoNT A injections at our University Eye Hospital, provided they agree to the therapy.

Diagnosis of BEB was based on anamnestic and clinical parameters, and the severity of the disease was assessed clinically, usually using various scales [27]. Since not all patients were assessed with the same scales over time, we refrained from analyzing these data. BoNT-A injections were administered periorbital into the M. orbicularis oculi in all patients. This could be supplemented individually by injections above the forehead and in the cheek and platysma. Since 1985, the BoNT-A drugs Botox (Onabotulinumtoxin A; Allergan Pharmaceuticals, Dublin, Ireland), Xeomin (Incobotuliumtoxin A; Firma Merz Pharma GmbH & Co., Frankfurt am Main, Germany), and Dysport (Abobotulinumtoxin A; Ipsen Biopharm Ltd., Wrexham, UK) were used at our clinic according to individual patient-related aspects and to their availability. For patients referred to us from other centers or treated at other centers in the interim, demographics and baseline parameters as well as data on injections performed at our department, were included in the analysis.

Since the introduction of BoNT-A treatment in 1985 in our hospital, BoNT-A injections for BEB treatment in our hospital are performed following a standardized treatment scheme. Moreover, data are recorded with a standardized documentation form. In addition, as part of standardized treatment, patients are specifically asked at re-presentation about subjectively perceived effect duration and adverse effects, with effect duration defined as the time from injection to the day of recurrence of symptoms reported by the patient. Data collected included demographics, diagnosis, number of injections, injection points, type of BoNT-A, dosage in IU per injection, subjective effect duration in weeks since the last injection, and subjective adverse effects of the last injection. The effect duration–dose quotient was determined. Long-term patients were defined as those who had received at least 21 injections, so complete data from at least 20 injections were documented and analyzed. Although botulinum toxin injections were usually administered symmetrically on both sides of the face, asymmetric injections also occurred in individual cases. Therefore, only data from one (the right) side of the face were considered for analysis. Data were analyzed using SAS 9.2 (SAS Institute Inc., Cary, NC, USA) and RStudio (R version 3.4.3; RStudio, Inc., Boston, MA, USA, available in the public domain at https://www.rstudio.com/, accessed on 29 December 2021) for graphical presentation. For comparisons between groups, the Pearson chi-square test (qualitative data), the Mann–Whitney–Wilcoxon test, or *t*-test (quantitative data) were used. Descriptive statistics are normally given for all patients and the subgroup of long-term patients. Nevertheless, the statistical tests formally compare the long-term patients with a complementary group of “non-long-term” patients. Mixed linear models were used for the analysis of long-term data. Time, represented by a consecutive number of injections, served as the influencing variable. The dependence of values collected for a patient over time was modeled using a patient-specific random intercept. This approach is designed to detect long-term changes as trends. More complex time courses may not be detected with this approach.

## Figures and Tables

**Table 1:** Demographics and treatment-associated parameters—comparison between Total Blepharospasm Group and Onabotulinumtoxin Long-Term Group (Ona Long-Term Group).

	Total Blepharospasm Group (*n* = 1940)	Ona Long Term Group (*n* = 234)
Gender, *n* (%) Female Male	1365 (70.4) 575 (29.6)	174 (74.4) 60 (25.6)
Age (years) first manifestation mean ± SD	58.0 ± 13.1	57.7 ± 11.9
Age (years) first visit mean ± SD	63.3 ± 11.9	62.3 ± 11.3
Frontalis-suspension, *n* (%)	130 (6.7)	34 (14.5)
Meige Syndrome, *n* (%)	591 (30.5)	51 (21.8)
Family history, *n* (%)	26 (1.3)	5 (2.1)
Patients with external treatment, *n* (%)	499 (25.7)	52 (22.2)
Number of injections/patient mean ± SD	17.5 ± 23.6	45.4 ± 22.9
Treatment duration (years) mean ± SD (range)	4.9 ± 6.2 (0.0–30.0)	12.6 ± 5.4 (2.9–30.0)
Dose of last analyzed injection (IU), mean ± SD	19.8 ± 7.6	22.7 ± 8.3

**Table 2:** Overview of long-term studies on BEB treatment with onabotulinum toxin A.

Reference	No. of Patients with BEB	Duration of Treatment (Years) Mean ± SD (Range)	Total Number of Injections	Number of Injections/Patient Mean ± SD	Subjective Effect Duration (Weeks) Mean ± SD	Definition of “Long-Term”
Present study	1940 (overall BoNT-A) 234 (Ona Long Term)	4.9 ± 6.2 (0.0–30.0) 12.6 ± 5.4 (2.9–30.0)	33,933 BoNT-A 10,632 Ona	17.5 ± 23.6 45.4 ± 22.9	10.3 ± 12.1 * 8.8 ± 0.3	Up to 30 Years TD ≥ 21 injections
Kollewe et al., 2015 [16]	288 (overall BoNT-A) 128 (Ona)	11.2 ± 4.1 (2.0–21.0) 10.3 ± 4.9 (2.0–21.0)	10,701 BoNT-A 4974	n.a. n.a.	10.2 ± 3.5 10.8 ± 3.3	Up to 21 years; ≥8 injections
Ababneh et al., 2014 [14]	21	14.5 ± 3.1 (10.0–19.0)	n.a. for BEB patients	49.6 ± 19.9	11.5 ± 7.2 (first year) 12.7 ± 5.2 (last year)	Up to 19 years /patients treated > 10 years
Bentiviglio et al., 2009 [19]	128 (overall BoNT-A)	n.a.	1341 (BoNT-A) 1009 (Ona)	10.6 ± 8.9 (BoNT-A)	9.5 ± 5.7	≥2 injections up to 15 years of treatment

BoNT-A, botulinumtoxin A; Ona, Onabotulinumtoxin A; n.a., data not available; TD, treatment duration; * of last analyzed injection.

## Data Availability

The data presented in this study are available on request from the corresponding author, as the original data is included in paper files.
